# Anti-GBM disease shortly after clinically presumed ANCA-associated vasculitis in an 85-year-old patient: a case report and literature review

**DOI:** 10.3389/fneph.2026.1967122

**Published:** 2026-09-28

**Authors:** Hiroki Ito, Hiyu Abe, Yuya Suzuki, Kentaro Yano, Fumiya Sato, Takaki Numahata, Risa Ishikawa, Akari Endo-Matsumoto, Keiji Matsumoto, Tsubasa Yuki, Ayaka Kamada, Hideaki Hashimoto, Go Anan, Katsuya Ishiyama, Takuo Hirose, Takefumi Mori

**Affiliations:** Division of Nephrology and Hypertension, Faculty of Medicine, Tohoku Medical and Pharmaceutical University, Sendai, Japan

**Keywords:** ANCA - associated vasculitis, anti-glomerular basal membrane nephritis, anti-glomerular basement antibody disease (anti-GBM disease), elderly, MPO- ANCA

## Abstract

**Introduction:**

Sequential emergence of anti-glomerular basement membrane (GBM) disease after anti-neutrophil cytoplasmic antibody (ANCA)-associated vasculitis (AAV) is rare.

**Case presentation:**

An 85-year-old woman presented with fever, edema, elevated myeloperoxidase (MPO)-ANCA, mild urinary abnormalities, markedly elevated urinary tubular injury markers, and negative serum anti-GBM antibodies measured by chemiluminescent enzyme immunoassay. Renal biopsy was not performed because of advanced age, severe dementia, antiplatelet therapy, and the family’s preference for non-invasive management. She was clinically presumed to have AAV and achieved apparent clinical remission with prednisolone alone, although MPO-ANCA remained positive. However, 2.5 months after the initial AAV diagnosis, she developed rapidly progressive kidney failure requiring dialysis, accompanied by severe hematuria, increased proteinuria, urinary casts, and markedly elevated anti-GBM antibodies. Clinically diagnosed anti-GBM disease was treated with methylprednisolone pulse therapy, prednisolone, plasma exchange, and hemodialysis. Although renal recovery was not achieved, anti-GBM antibodies became negative, and she remained clinically stable on maintenance hemodialysis for over 4 years while receiving low-dose prednisolone.

**Conclusion:**

This case suggests that anti-GBM disease can emerge shortly after apparent clinical remission of AAV. ANCA-mediated GBM injury and age-related GBM changes may contribute to subsequent anti-GBM autoimmunity, although these mechanisms remain hypothetical in this patient. Prompt anti-GBM antibody testing should be considered when a rapid, unexplained decline in kidney function occurs during or after AAV treatment.

## Introduction

The coexistence of anti-neutrophil cytoplasmic antibody (ANCA) and anti-glomerular basement membrane (GBM) antibodies, frequently described as “double-positive” disease, is a well-recognized clinical phenomenon that typically manifests as severe crescentic glomerulonephritis (1, 2). While most reported cases involve the simultaneous detection of both autoantibodies, the sequential appearance of these diseases—where one antibody follows the other with a distinct clinical interval—is rare (3, 4).

Sequential development of anti-GBM disease after established AAV has rarely been reported (3–12).

Here, we report an 85-year-old woman whose anti-GBM antibodies were initially negative by chemiluminescent enzyme immunoassay but became markedly elevated 2.5 months after the diagnosis of ANCA-associated vasculitis (AAV), during apparent clinical remission.

## Case description

### Initial presentation and diagnosis of AAV

An 85-year-old Japanese woman presented in November of Year X with several days of fever and bilateral lower-extremity edema. Her past medical history included a prior cerebral infarction, for which she was treated with aspirin (100 mg/day). She also had severe dementia, which substantially limited her ability to provide detailed medical information. There was no history of preceding infection.

On admission in November of Year X, physical examination revealed a body temperature of 37.8 °C, blood pressure of 142/84 mmHg, and a heart rate of 88 beats per minute. Mild pitting edema was noted in both lower extremities. There were no signs of skin rash, joint swelling, or macroscopic hematuria. Chest X-ray and chest computed tomography at the initial presentation showed a small pleural effusion without evidence of interstitial pneumonia or alveolar hemorrhage (**Figures 1A, B**). Abdominal computed tomography showed preserved renal morphology without apparent atrophy (**Figure 1C**). Laboratory investigations showed a serum creatinine (sCr) level of 0.97 mg/dL, cystatin C (CysC) of 2.09 mg/L, and a C-reactive protein (CRP) level of 7.32 mg/dL. Because of her advanced age and low body weight, creatinine-based eGFR may have overestimated kidney function because of reduced muscle mass; therefore, cystatin C-based eGFR was also evaluated. Immunological testing revealed a high titer of myeloperoxidase (MPO)-ANCA (206 U/mL), while anti-GBM antibodies were negative (< 2.0 U/mL; reference range, < 2.0 U/mL) (**Table 1**). MPO-ANCA and anti-GBM antibodies were measured at SRL, Inc. (Tokyo, Japan) by chemiluminescent enzyme immunoassay (CLEIA) using the STACIA MEBLux Test MPO-ANCA and STACIA MEBLux Test GBM, respectively (Medical & Biological Laboratories Co., Ltd., Japan), on the STACIA automated platform. Urinalysis demonstrated microscopic hematuria (5–9 red blood cells/high-power field) and proteinuria (urine protein-to-creatinine ratio: 0.62 g/gCr), with a markedly elevated urinary β2-microglobulin (uβ2MG) level (21,200 μg/L) (**Table 1**). Although a renal biopsy was not performed because of the patient’s advanced age, severe dementia, antiplatelet therapy, and the family’s preference for non-invasive management, she was clinically presumed to have MPO-ANCA-positive AAV. She had no asthma, eosinophilia, upper-airway involvement, pulmonary nodules, or other granulomatous manifestations. Based on mild hematuria and proteinuria, normoglycemic glycosuria, and markedly elevated urinary β2-microglobulin, the initial renal involvement was considered to include prominent tubular injury despite limited urinary evidence of glomerular involvement, with peritubular capillaritis (PTCitis) considered a possible underlying mechanism.

**Figure 1:**
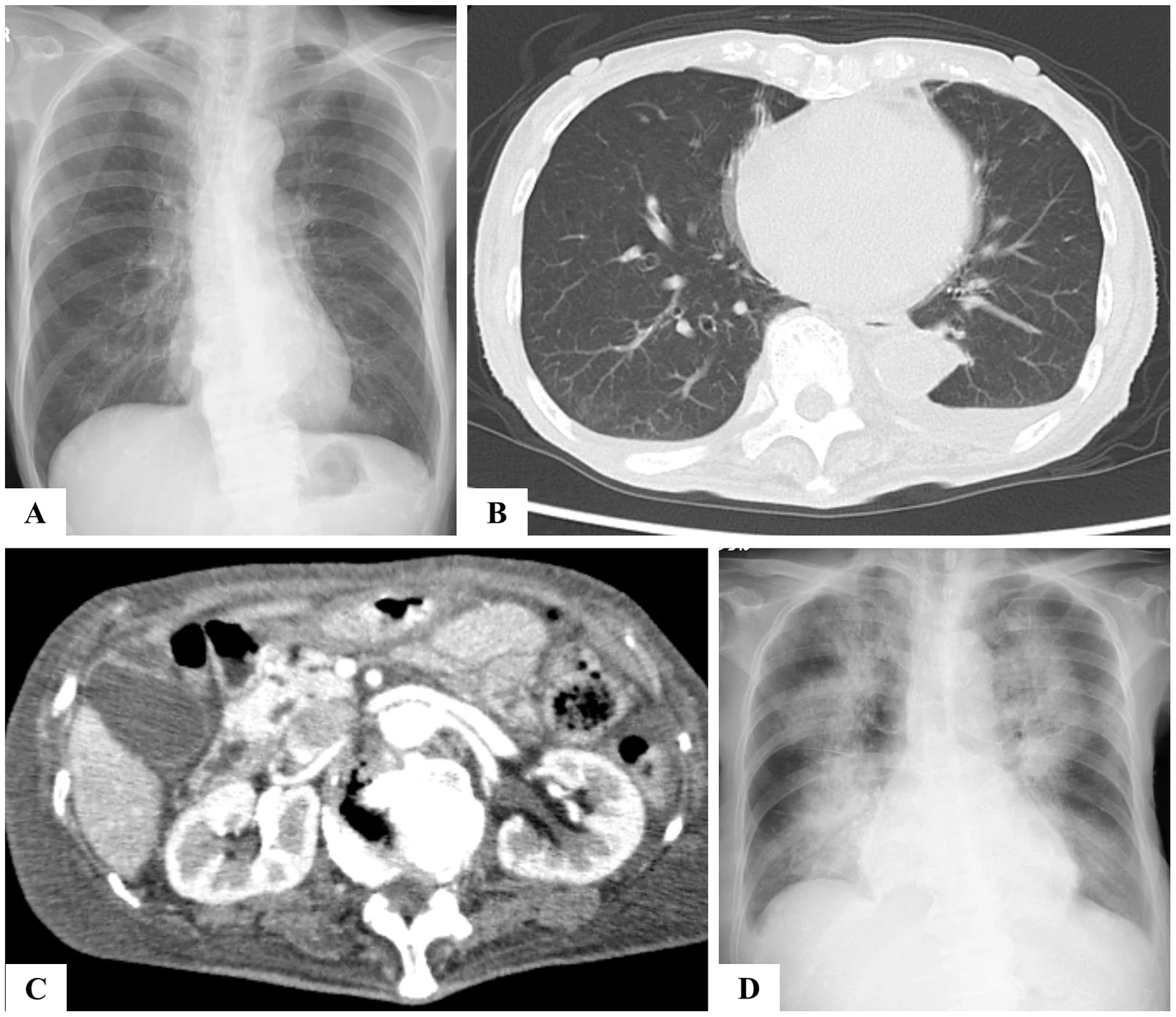
Radiological findings of the patient. **(A)** Chest X-ray and **(B)** chest computed tomography at the initial presentation show a small pleural effusion without evidence of interstitial pneumonia or alveolar hemorrhage. **(C)** Abdominal computed tomography at the initial presentation shows preserved renal morphology without apparent atrophy. **(D)** Chest X-ray at the second admission shows pulmonary edema and bilateral pleural effusion.

**Table 1:** Clinical and laboratory profile of the patient.

Laboratory parameter	Initial visit November of Year X	Pre-anti-GBM onset December of Year X	Anti-GBM onset January of Year X + 1
Hematology			
White blood cell (/µL)	7900	7300	12700
Red blood cell (×10^6^/µL)	2.58	3.19	2.20
Hemoglobin (g/dL)	7.6	10.1	6.8
Platelet (×10^3^/µL)	348	218	200
Blood chemistry			
AST (U/L)	21	20	13
ALT (U/L)	9	12	12
LDH (U/L)	127	151	168
Albumin (g/dL)	2.4	3.6	2.3
BUN (mg/dL)	22	18	109
Creatinine (mg/dL)	0.97	0.69	5.91
CRP (mg/dL)	7.32	1.35	5.75
eGFR (mL/min/1.73m^2^)	41.6	60.3	5.8
Cystatin C (mg/L)	2.09	1.18	N/A
eGFR-cystatin C (mL/min/1.73m^2^)	24.7	50.3	N/A
HbA1c (%)	5.6	5.5	5.3
BNP (pg/mL)	119.7	N/A	334.2
Immunology			
IgG (mg/dL)	1872	1214	1444
IgA (mg/dL)	274	N/A	237
IgM (mg/dL)	167	N/A	170
ANA	<40	N/A	N/A
CH50 (U/mL)	58.2	N/A	55.6
C3 (mg/dL)	96	N/A	94
C4 (mg/dL)	18	N/A	23
PR3-ANCA (U/mL)	<1.0	N/A	<1.0
MPO-ANCA (U/mL)	206	181	88.4
Anti-GBM antibody (U/mL)	<2.0	N/A	317
Urinalysis			
Sugar (dipstick)	1+	(–)	(±)
Protein (dipstick)	1+	(–)	2+
Occult blood (dipstick)	1+	±	3+
NAG (IU/L)	34	6.1	13.2
uβ2MG (µg/L)	21200	3230	11100
RBC (**/**HPF)	5-9	1-4	>100
Urine protein-to-creatinine ratio (g/gCr)	0.62	0.29	2.72
Cast	Granular, Epithelial, Hyaline	–	Red blood cell, White blood cell, Granular, Waxy, Epithelial

AST, aspartate aminotransferase; ALT, alanine aminotransferase; LDH, lactate dehydrogenase; BUN, blood urea nitrogen; CRP, C-reactive protein; eGFR, estimated glomerular filtration rate; HbA1c, hemoglobin A1c; BNP, B-type natriuretic peptide; IgG, immunoglobulin G; IgA, immunoglobulin A; IgM, immunoglobulin M; ANA, antinuclear antibody; C3, complement 3; C4, complement 4; CH50, complement hemolytic activity; MPO-ANCA, myeloperoxidase anti-neutrophil cytoplasmic antibody; PR3-ANCA, proteinase 3 anti-neutrophil cytoplasmic antibody; GBM, glomerular basement membrane; NAG, N-acetyl-beta-D-glucosaminidase; uβ2MG, urinary β2-microglobulin; RBC, red blood cell; HPF, high-power field; N/A, not available.

Creatinine-based eGFR was calculated using the Japanese Society of Nephrology equation: eGFR = 194 × serum creatinine^-1.094 × age^-0.287 × 0.739 (if female). Cystatin C-based eGFR was calculated as follows: eGFR-cystatin C = 104 × cystatin C^-1.019 × 0.996^age × 0.929 (if female) − 8.

The patient was started on oral prednisolone (PSL) at 40 mg/day (1 mg/kg) for 6 days, followed by 30 mg/day for 6 days, 20 mg/day for 10 days, and 10 mg/day for 20 days. She was discharged while receiving PSL 10 mg/day, which was subsequently reduced to 7.5 mg/day. Given her advanced age, severe dementia, relatively mild renal presentation, and absence of pulmonary hemorrhage or other life-threatening manifestations, corticosteroid monotherapy was selected to minimize the risk of treatment-related adverse events. By December of Year X, sCr, CysC, and uβ2MG levels had decreased to 0.69 mg/dL, 1.18 mg/L, and 3,230 μg/L, respectively, and hematuria had resolved (**Table 1**). Because of this favorable clinical response and concern regarding treatment-related toxicity, PSL was tapered relatively rapidly to 7.5 mg/day by late December. At that time, she appeared to be in clinical remission, with no signs of vasculitis flare; however, her MPO-ANCA titer remained high at 181 U/mL (**Table 1**).

### Development of anti-GBM disease

In January of Year X + 1, while receiving PSL at 7.5 mg/day, the patient suddenly developed marked lower-extremity edema and dyspnea. Her body weight had increased by 9.3 kg over approximately 7 weeks, from 37.1 kg at the previous discharge to 46.4 kg at readmission, indicating severe fluid overload. Chest radiography revealed pulmonary edema and bilateral pleural effusion (**Figure 1D**). Laboratory investigations confirmed a rapid decline in renal function, with sCr rising to 5.91 mg/dL, BUN reaching 109 mg/dL, and CRP at 5.75 mg/dL (**Table 1**). Urinalysis demonstrated severe hematuria (>100 red blood cells/high-power field), increased proteinuria (2.72 g/gCr), and various types of casts, including red blood cell and granular casts (**Table 1**). Serum anti-GBM antibodies, measured using the same CLEIA method, had increased to 317 U/mL, while MPO-ANCA remained positive at 88.4 U/mL (**Table 1**). A renal relapse of AAV was considered; however, the marked anti-GBM antibody elevation, together with rapidly progressive kidney failure and active urinary sediment, strongly supported newly developed anti-GBM disease. Although MPO-ANCA remained positive, its titer had decreased from the initial value, and no overt extrarenal vasculitic manifestations were observed. Because renal biopsy was not performed, concomitant AAV renal relapse could not be completely excluded. Emergency hemodialysis was initiated via a femoral vein catheter for refractory fluid overload and uremic symptoms.

### Treatment and follow-up

She received methylprednisolone pulse therapy (500 mg/day for 3 days), followed by oral PSL at 20 mg/day (0.5 mg/kg), together with five sessions of plasma exchange. After five sessions, the anti-GBM antibody titer had decreased to a nearly undetectable level, whereas anuria persisted without evidence of kidney recovery; therefore, no further plasma exchange was performed. Three days after the clinical diagnosis of anti-GBM disease, she developed fever with leukocytosis (26,500/μL), elevated CRP (14.29 mg/dL), and procalcitonin (5.81 ng/mL). Blood cultures were negative, whereas urine culture grew *Escherichia coli* (3+), consistent with a urinary tract infection. Ceftriaxone was administered for 10 days, after which the white blood cell count decreased to 9,300/μL and CRP to 0.90 mg/dL. Cyclophosphamide and rituximab were withheld because of the active *Escherichia coli* urinary tract infection and the high risk of treatment-related toxicity associated with her advanced age, severe dementia, and frailty. Despite corticosteroid therapy, plasma exchange, and hemodialysis, there were no signs of recovery in renal function, and she was transitioned to maintenance hemodialysis (**Figure 2**).

**Figure 2:**
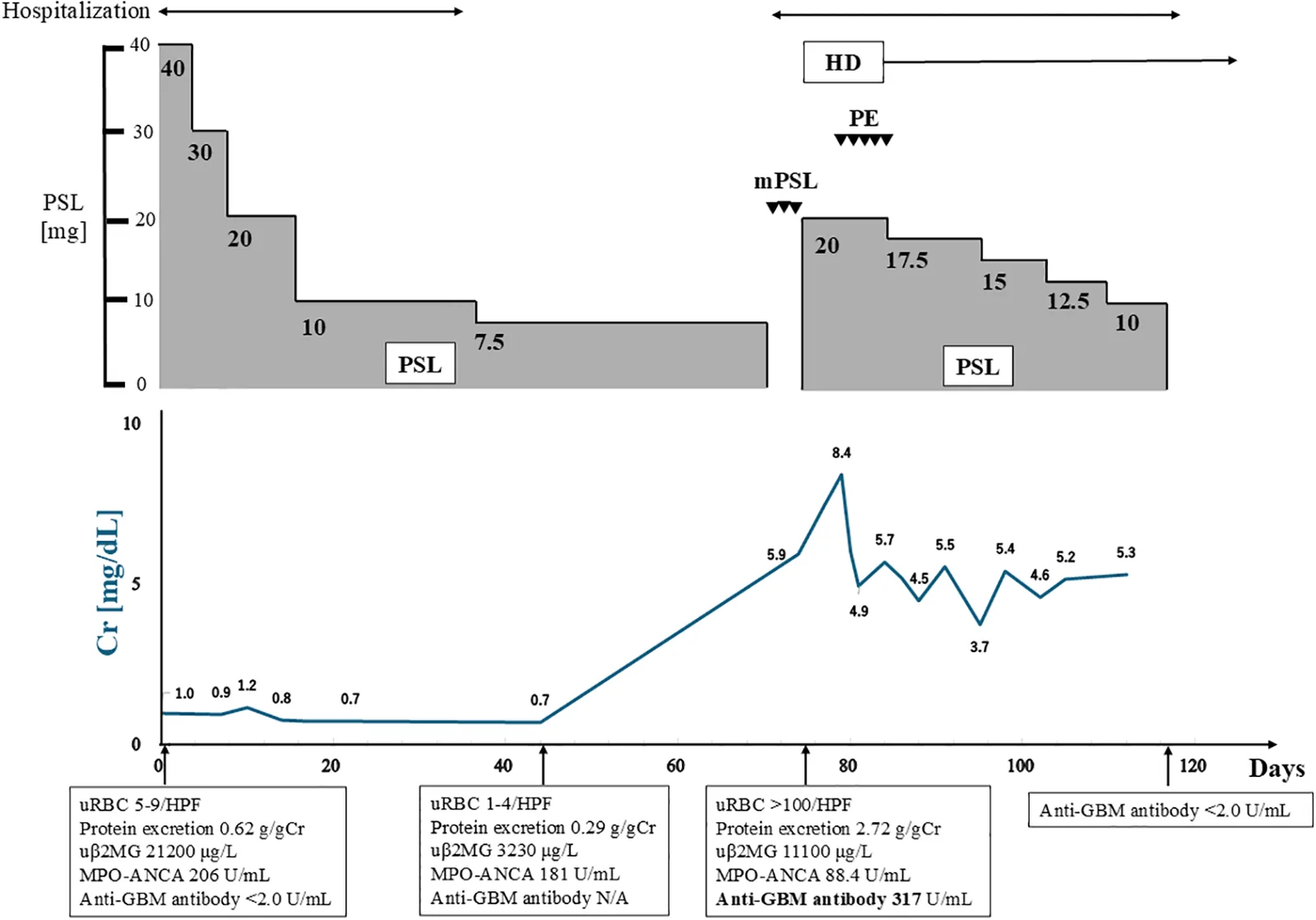
Clinical course and laboratory findings during the two hospitalizations. PSL, prednisolone; mPSL, methylprednisolone; PE, plasma exchange; HD, hemodialysis; Cr, serum creatinine; uRBC, urinary red blood cells; uβ2MG, urinary β2-microglobulin; MPO-ANCA, myeloperoxidase anti-neutrophil cytoplasmic antibody; GBM, glomerular basement membrane.

After the transition to maintenance hemodialysis, PSL was tapered to 2.5 mg/day within 6 months because of her advanced age and dialysis dependence. During 4 years of follow-up, she had no relapse of AAV or anti-GBM disease. Anti-GBM antibodies remained negative, while MPO-ANCA remained weakly positive at approximately 10–20 U/mL without elevated inflammatory markers or new systemic symptoms.

## Discussion

The sequential development of anti-GBM disease following apparent clinical remission of AAV is uncommon, with only a few cases reported in the literature (3–12) (**Table 2**). ANCA positivity is relatively common among patients with anti-GBM disease; a recent meta-analysis of 47 studies including 2,830 patients reported a pooled ANCA positivity rate of 27.4% (13). However, most available cohorts address simultaneous ANCA/anti-GBM double positivity, whereas the present literature review specifically focuses on cases in which established AAV preceded anti-GBM disease by a distinct clinical interval. We searched PubMed/MEDLINE, CiNii Research, J-STAGE, and Ichushi-Web through September 5, 2026, using English and Japanese terms related to AAV, ANCA, MPO-ANCA, and anti-GBM disease. We included cases in which established AAV preceded anti-GBM disease by a distinct clinical interval and excluded simultaneous double-positive and reverse-sequence cases. Among the published cases identified in our literature review, our patient was the oldest and had the shortest interval between AAV and anti-GBM disease, with onset occurring only 2.5 months after the initial AAV diagnosis. Unlike simultaneous “double-positive” disease, the delayed appearance of anti-GBM antibodies may reflect epitope spreading (5, 14). In this proposed mechanism, inflammation induced by AAV may disrupt the quaternary structure of the type IV collagen network within the GBM, thereby exposing “cryptic” epitopes of the α3(IV)NC1 domain that are normally sequestered from the immune system (14).

**Table 2:** Reported cases of anti-GBM disease developing sequentially after established ANCA-associated vasculitis.

Item	This case	Case 1 (3)	Case 2 (3)	Case 3 (6)	Case 4 (4)	Case 5 (7)	Case 6 (9)	Case 7 (8)	Case 8 (5)	Case 9 (10)	Case 10 (11)	Case 11 (12)
Age at AAV diagnosis (years)/Sex	**85/female**	60/male	23/female	70/male	72/male	54/male	74/male	68/male	72/female	62/female	53/male	73/male
Prior AAV (subtype)	MPO-AAV	EGPA	GPA	GPA	MPA	AAV	AAV	GPA	AAV	MPO-AAV	MPA	MPO-AAV
ANCA type at AAV onset (MPO/PR3/other)	MPO-ANCA	MPO-ANCA	MPO-ANCA	NR	MPO-ANCA	MPO-ANCA	MPO-ANCA	MPO-ANCA	MPO-ANCA	MPO-ANCA	MPO-ANCA	MPO-ANCA
Anti-GBM antibody at AAV onset (positive/negative; value if available)	Negative	NT	NT	NR	Negative	Negative	NR	Negative	Negative	Negative	NR	NR
sCr at AAV onset (mg/dL)	0.97	0.8	0.9	NR	1.7	2.0	NR	6.3	2.89	NR	NR	2.3
Time to anti-GBM onset	2.5 months	3 years	3 years	6 months	3 years	9 years	4 years	2.6 years	1 year	9 months	1 year	22 months
ANCA status at anti-GBM onset	Positive	Positive	Negative	NR	Positive	Negative	Positive	Positive	Negative	Negative	Positive	Positive
Anti-GBM antibody at onset	317 U/mL	>200 IU/mL	>200 IU/mL	>1:33,000 (titer)	NR	122.2 U/L	140 EU	138 U/mL	>680 IU/mL	Strongly positive; value NR	Positive; value NR	50 EU
sCr at anti-GBM onset (mg/dL)	5.91	8.9	5.2	16.3	NR	11.9	16.9	12.0	6.29	NR	NR	5.3
Renal prognosis (dialysis dependent/CKD/recovery)	Dialysis dependent	Dialysis dependent	CKD	Dialysis dependent	Dialysis dependent	Dialysis dependent	Dialysis dependent	Dialysis dependent	Dialysis dependent	NR	NR	Dialysis dependent
Survival outcome	Survival	Survival	Survival	Survival	Survival	Death	Death	Survival	Survival	NR	Death	Death
Follow-up after anti-GBM onset (duration, endpoint)	4 years	Duration NR; ESKD on dialysis	≥6 months; endpoint stable eGFR 52 (sCr 1.3)	≥5 months	NR	26 days; death	6 months; death (pulmonary infection)	2 years; ESKD on dialysis	2 years; ESKD on dialysis	NR	Duration NR; death from complications	3 months; death from pancreatitis and multiple organ failure associated with TTP
Relapse after anti-GBM onset (AAV relapse/anti-GBM relapse; Yes/No)	No	No	Yes (anti-GBM relapse)	NR	NR	NR	NR	NR	No	NR	NR	No

GBM, glomerular basement membrane; ANCA, anti-neutrophil cytoplasmic antibody; AAV, ANCA-associated vasculitis; MPA, microscopic polyangiitis; MPO, myeloperoxidase; PR3, proteinase 3; EGPA, eosinophilic granulomatosis with polyangiitis; GPA, granulomatosis with polyangiitis; sCr, serum creatinine; CKD, chronic kidney disease; NR, not reported; NT, not tested; ESKD, end-stage kidney disease.

At AAV onset, mild hematuria and proteinuria contrasted with markedly elevated urinary β2-microglobulin and normoglycemic glycosuria, suggesting prominent tubular injury despite limited urinary evidence of glomerular involvement. Urinary low-molecular-weight protein excretion has been associated with PTCitis in AAV (15), and AAV can present with tubulointerstitial injury without substantial glomerular lesions (16). PTCitis may therefore have contributed to the tubular injury in this case, although this could not be confirmed without renal histology.

Anti-GBM disease has been reported in only approximately 40 patients with IgA nephropathy, the most common primary glomerulonephritis worldwide (17). Because IgA nephropathy primarily affects the mesangium, it may be less likely than AAV to directly injure the GBM. In AAV, ANCA-activated neutrophils release reactive oxygen species and proteolytic enzymes, which may damage the GBM and promote exposure of cryptic antigens (18, 19). Age-related GBM alterations may have increased susceptibility to ANCA-mediated injury, although this remains speculative without histological or molecular evidence. Aging kidneys show GBM folding and thickening, altered glycosaminoglycan sulfation, and changes in type IV collagen and laminins (20–22). These changes may have lowered the threshold for GBM injury, although the extent of their contribution in this patient is unknown. A recent review recognizes anti-GBM disease in older individuals as a clinical phenotypic variant and ANCA-associated anti-GBM disease as an overlapping autoimmune syndrome (23). Thus, both epitope spreading and age-related GBM susceptibility should be regarded as plausible hypotheses rather than mechanisms directly demonstrated in this patient.

Although sCr and CysC improved and hematuria resolved within 1.5 months of the initial presentation, MPO-ANCA remained high at 181 U/mL. Because there was no overt clinical evidence of active vasculitis, the patient was considered to have achieved apparent clinical remission despite persistent seropositivity. In retrospect, the persistently high MPO-ANCA level during the relatively rapid PSL taper raises the possibility that subclinical ANCA-mediated inflammation was incompletely suppressed, although persistent MPO-ANCA positivity alone does not establish ongoing vasculitic activity. Such inflammation, if present, could have promoted GBM damage and exposure of cryptic epitopes, allowing anti-GBM disease to emerge without an overt AAV flare. This proposed mechanism remains hypothetical in the present patient. Whether a slower glucocorticoid taper or additional immunosuppressive therapy would have prevented this transition remains unknown; therefore, the temporal association should not be interpreted as proof of causality. Crucially, anti-GBM antibodies can emerge in the absence of overt clinical relapse of the primary vasculitis; as seen in our case and others, secondary anti-GBM disease can develop during apparent clinical remission or under maintenance immunosuppressive therapy (**Table 2**) (1, 3–12). This process may promote loss of immunological tolerance even in the absence of overt systemic vasculitis activity (14, 18), potentially facilitated by age-related changes. Previously reported cases involved patients aged 23–74 years and intervals of 6 months to 9 years (**Table 2**) (3–12).

Current KDIGO guidance recommends plasma exchange until circulating anti-GBM antibodies are no longer detectable, together with glucocorticoids and cyclophosphamide; recent treatment guidance similarly emphasizes individualized treatment when standard immunosuppression is contraindicated (24, 25). In our patient, anti-GBM antibodies became negative (<2.0 U/mL) after five plasma exchanges, and no further exchange was performed. Cyclophosphamide was withheld because of the active *Escherichia coli* urinary tract infection and concerns regarding treatment-related toxicity in the setting of advanced age, severe dementia, and frailty; rituximab was likewise not pursued as an alternative.

In this patient, immunosuppression was cautiously reduced after the transition to maintenance hemodialysis, without subsequent clinical relapse over 4 years. Although this favorable course is noteworthy, it should not be interpreted as evidence supporting routine de-escalation of immunosuppressive therapy in other patients.

The principal limitation is the absence of renal histology because of the patient’s advanced age, severe dementia, aspirin use, and the family’s preference for non-invasive management. Thus, neither diagnosis was histologically confirmed, and prognostic features such as the percentage of normal glomeruli, an important predictor of kidney recovery even in dialysis-dependent anti-GBM disease, could not be assessed (26, 27). Although serum anti-GBM antibody assays have high diagnostic accuracy, a meta-analysis reported a pooled sensitivity of 93% and specificity of 97% (28); therefore, the initial result of <2.0 U/mL cannot completely exclude a very low circulating antibody level. Nevertheless, the subsequent increase from <2.0 to 317 U/mL using the same CLEIA method, together with severe hematuria, increased proteinuria, and urinary casts, fulfilled the Japanese diagnostic criteria for anti-GBM glomerulonephritis (29).

This case suggests that anti-GBM disease can emerge shortly after apparent clinical remission of AAV in an 85-year-old patient. ANCA-mediated GBM injury and age-related GBM changes may contribute to subsequent anti-GBM autoimmunity, although these mechanisms remain hypothetical in this patient. Clinicians should consider prompt anti-GBM antibody testing when a rapid, unexplained decline in kidney function occurs during or after AAV treatment.

## Patient perspective

A patient perspective could not be obtained because of the patient’s severe dementia.

## Data Availability

The original contributions presented in the study are included in the article/supplementary material. Further inquiries can be directed to the corresponding author.
